# Evaluation of Macular Retinal Ganglion Cell-Inner Plexiform Layer Thickness after Vitrectomy with Internal Limiting Membrane Peeling for Idiopathic Macular Holes

**DOI:** 10.1155/2014/458631

**Published:** 2014-07-07

**Authors:** Alfonso L. Sabater, Álvaro Velázquez-Villoria, Miguel A. Zapata, Marta S. Figueroa, Marta Suárez-Leoz, Luis Arrevola, María-Ángeles Teijeiro, Alfredo García-Layana

**Affiliations:** ^1^Department of Ophthalmology, Clínica Universidad de Navarra, 31008 Pamplona, Spain; ^2^Department of Ophthalmology, Hospital Universitario Vall d'Hebrón, 08035 Barcelona, Spain; ^3^Red Temática de Investigación Cooperativa en Oftalmología (RETICS-Oftared RD12/0034), Instituto de Salud Carlos III, Madrid, Spain; ^4^Department of Ophthalmology, Hospital Universitario Ramón y Cajal, 28034 Madrid, Spain; ^5^Clínica Oftalmológica Suárez Leoz, 28010 Madrid, Spain; ^6^Clínica Baviera, 28046 Madrid, Spain

## Abstract

*Purpose*. To evaluate macular retinal ganglion cell-inner plexiform layer (GCIPL) thickness changes after Brilliant Blue G-assisted internal limiting membrane peeling for idiopathic macular hole repair using a high-resolution spectral-domain optical coherence tomography (SD-OCT). *Methods*. 32 eyes from 32 patients with idiopathic macular holes who underwent vitrectomy with internal limiting membrane peeling between January 2011 and July 2012 were retrospectively analyzed. GCIPL thickness was measured before surgery, and at one month and at six months after surgery. Values obtained from automated and semimanual SD-OCT segmentation analysis were compared (Cirrus HD-OCT, Carl Zeiss Meditec, Dublin, CA). *Results*. No significant differences were found between average GCIPL thickness values between preoperative and postoperative analysis. However, statistical significant differences were found in GCIPL thickness at the temporal macular quadrants at six months after surgery. Quality measurement analysis performed by automated segmentation revealed a significant number of segmentation errors. Semimanual segmentation slightly improved the quality of the results. *Conclusion*. SD-OCT analysis of GCIPL thickness found a significant reduction at the temporal macular quadrants at 6 months after Brilliant Blue G-assisted internal limiting membrane peeling for idiopathic macular hole.

## 1. Introduction

Nowadays, one of the most common surgical procedures for idiopathic macular hole (IMH) management is based on vitrectomy with internal limiting membrane (ILM) peeling [1–3]. Different vital dyes such as indocyanine green (ICG) or Brilliant Blue G (BBG) and other substances such as triamcinolone acetonide (TA) have been used to assist in peeling of the ILM of the neuroretina [4–6]. However, several authors have reported histological and functional damage to the retina after IMH surgery with ICG-assisted ILM peeling [3, 7–10]. In contrast, BBG or TA appears to be safer alternatives for ILM peeling [6, 11, 12].

On the other hand, ILM peeling itself may induce visible changes of the inner retinal surface, although no changes in retinal nerve fiber layer (RNFL) thickness have been detected [13]. However, there is some controversy about the effect that BBG-assisted ILM peeling has on the retinal ganglion cell complex (RGCC) [14, 15]. Moreover, a recent study has suggested that ILM peeling may reduce retinal sensitivity and increase the incidence of microscotomas [16].

To our knowledge, only a few groups have evaluated the effect of ILM peeling on the RGCC after idiopathic macular hole surgery using the RTVue-100 SD OCT (Optovue, Fremont, CA, USA) with different results [14, 15, 17]. In the current study, we evaluated for the first time the capacity of the new ganglion cell analysis (GCA) software of the Cirrus HD-OCT (Carl Zeiss Meditec, Dublin, CA) to analyze the retinal ganglion cell-inner plexiform layer (GCIPL) after BBG-assisted internal limiting membrane peeling for idiopathic macular hole surgery. Additionally, we evaluated how BBG-assisted ILM peeling affects the macular and average GCIPL thickness at 1 and 6 months after macular hole surgery with this new software.

## 2. Materials and Methods

**Figure 1:**
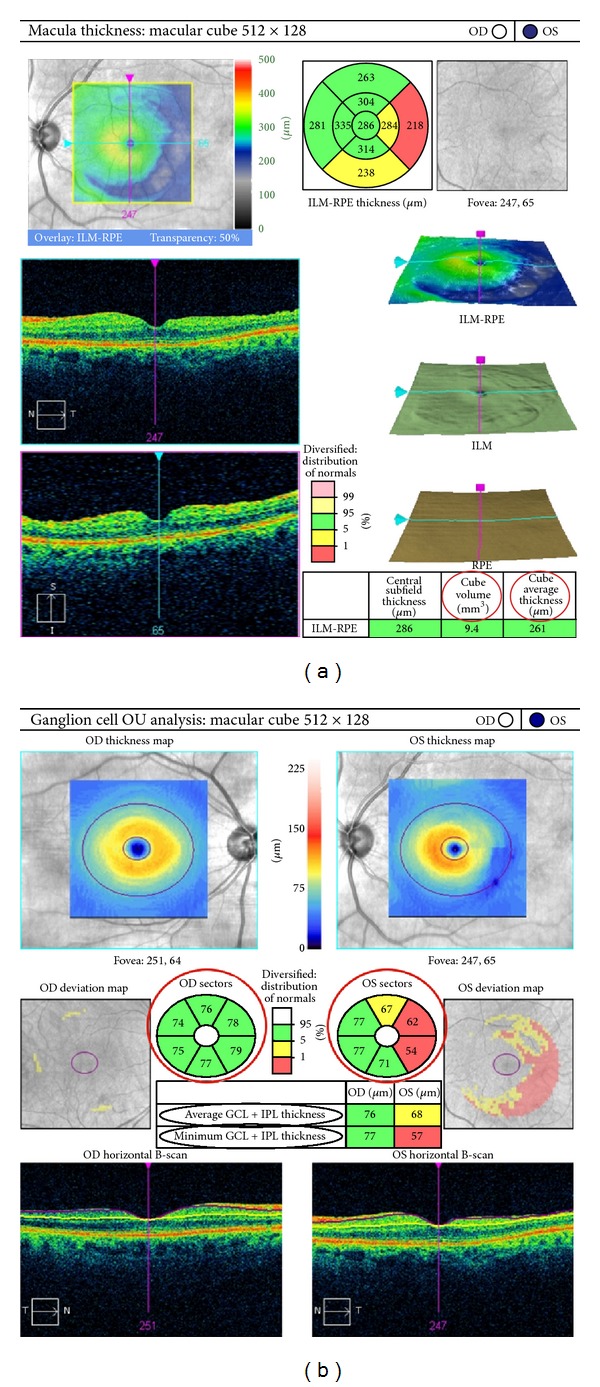
Macular analysis of the left eye and ganglion cell analysis of both eyes of a 78-year-old woman at 6 months after vitrectomy with Brilliant Blue G-assisted internal limiting membrane peeling for idiopathic macular hole. (a) OCT cross-section analysis showing cube volume and cube thickness. (b) Average, minimum, and sectorial macular thickness of the ganglion cell-inner plexiform layer of both eyes. A macular temporal defect may be appreciated in the left eye.

This study was a multicenter (*n* = 5), retrospective, and observational study of 32 patients. The institutional review board approval of every center was obtained. All of the patients underwent a vitrectomy associated with BBG-assisted peeling of the retinal ILM as a consequence of an IMH between January 2011 and July 2012. Seven patients were excluded for the following reasons: a history of glaucoma (1), failure to correctly identify the limits of the GCIPL by the ganglion cell analysis software by automated or semimanual segmentation (4), or macular holes greater than the central area of analysis where the GCA software does not measure the GCIPL thickness (2) (Figure 1). Therefore, a total of 25 eyes of 25 patients were included in this study.

Demographic information collected from the clinical chart included patient age, sex, combined cataract surgery, macular hole stage, preoperative best-corrected visual acuity (BCVA), postoperative BCVA, intraocular hypertension after surgery (>25 mmHg), history of glaucoma, and failure to close the macular hole. Best-corrected visual acuity was measured using a decimal visual acuity chart, and the decimal visual acuity was converted to the logarithm of the minimum angle of resolution (logMAR) units for statistical analysis.

Three-dimensional cube OCT data were obtained with the Cirrus HD-OCT device using the Macular Cube 200 × 200 scan protocol. This protocol performs 200 horizontal B-scans comprising 200 A-scans per B-scan over 1024 samples within a cube measuring 6 × 6 × 2 mm. The GCA software (6.0 version) evaluates the thickness of the ganglion cell plus inner plexiform layers. The average, minimum, and sectorial thicknesses of the GCIPL are measured in an elliptical annulus (vertical inner and outer radius of 0.5 mm and 2.0 mm; horizontal inner and outer radius of 0.6 and 2.4 mm, resp.) around the fovea. In order to avoid segmentation errors, OCT measurements with signal strength (SS) below 5 were excluded (0: lowest SS; 10: highest SS).

All OCT images were obtained by experienced clinical technicians. Eyes were dilated with tropicamide 1% and phenylephrine 2.5%. Average GCIPL thickness, macular cube average thickness (MCAT), and macular cube volume (MCV) values of the patients included in this study were measured preoperatively, at 1 and at 6 months after macular hole surgery by scanning with the Cirrus HD-OCT system (Carl Zeiss Meditec, Dublin, CA) (Figure 1).

The main outcome measure was the comparison of average GCIPL thickness preoperatively and at 6 months after macular hole surgery with BBG-assisted ILM peeling. Comparison of MCAT and MCV preoperatively and at 6 months after macular hole surgery with ILM peeling was the secondary outcome measures. Moreover, all values were obtained at 1 month after surgery. Average, minimum, and sectorial (superior, inferior, superonasal, inferonasal, superotemporal, and inferotemporal) GCIPL thickness values were obtained and compared in every patient preoperatively and at 1 and 6 months after surgery (Figure 1).

Each GCIPL scan was evaluated in order to identify how many cases had a greater GCIPL thickness after surgery compared to before. This data was studied to evaluate the quality of the measurements, as the real GCIPL thickness should not be higher in the postoperative period.

**Figure 2:**
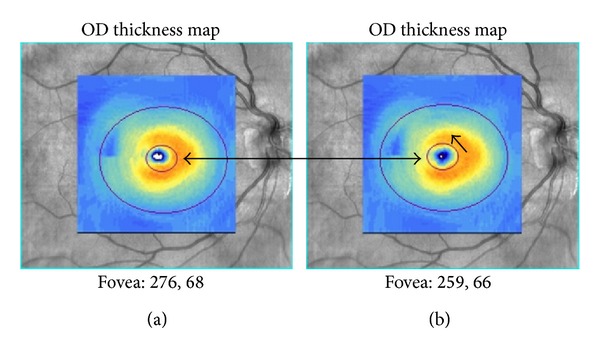
This example shows the relocation of the area of analysis (semimanual segmentation). The center was manually displaced following the direction of the black arrow (b).

A comparison between preoperative and postoperative macular GCIPL thickness values was also performed by semimanual segmentation. The Cirrus HD-OCT (Carl Zeiss Meditec, Dublin, CA) GCIPL analysis software is not capable of real manual segmentation of the macular layers, but it does allow relocation of the area of analysis (Figure 2). This procedure was performed in every scan by an experienced clinical technician in order to improve the quality of the measurements by repositioning the area of analysis in the real center of the fovea.

Surgery was performed using a standard 23- or 25-gauge 3-port pars plana vitrectomy. The infusion cannula was placed in the inferotemporal quadrant. If the posterior hyaloid was still attached to the optic disc, its detachment was induced by suction with the vitrectomy probe. A volume of 0.1 mL BBG (Fluoron GmbH, Ludwigsfeld, Germany) at a concentration of 0.25 mg/mL was injected into the vitreous cavity over the posterior pole for 30 seconds. The ILM was grasped at the temporal quadrant and peeled off with forceps in an area of 2-disc diameter around the macular hole. Fluid-air exchange and intraocular gas tamponade with SF6 at 20% were performed. After surgery, patients were asked to remain in a facedown position for at least 50 minutes per hour for four days. In 12 patients, the crystalline lens was removed by phacoemulsification followed by intraocular lens implantation before pars plana vitrectomy. A topical beta blocker (timolol maleate 0.5% BID) was routinely used to prevent postoperative intraocular pressure (IOP) rise.

The differences in the OCT values between the preoperative time and at 1 and at 6 months after surgery were analyzed using the paired *t*-test. The descriptive statistics are expressed as the means, standard deviations (SDs), and percentages. Visual acuity data were converted to the logarithm of the minimal angle of resolution (logMAR). Statistical analysis was performed using SPSS, version 15.0 (SPSS Inc., Chicago, IL). A *P* value of ≤0.05 was considered significant.

## 3. Results and Discussion

The study sample was comprised of 25 eyes of 25 participants (mean age 70.48 ± 8.66 years old, range: 49–82). Mean preoperative and postoperative (6 months) BCVA were 0.7 ± 0.32 logMAR units and 0.34 ± 0.32 logMAR units, respectively. The rate of closure of macular holes by OCT evaluation was 100% at 1 and 6 months after surgery. Twelve patients underwent a combined cataract surgery with pars plana vitrectomy. None of the patients had a postoperative retinal detachment. There was no recorded incidence of increased postoperative IOP above 25 mmHg. Demographic data are shown in Table 1.

Average MCV was 10.22 ± 0.81 *μ*m at the preoperative period, 10.03 ± 1.06 *μ*m at 1 month after surgery, and 9.85 ± 0.95 *μ*m at 6 months after surgery. Additionally, MCAT was 283.92 ± 21.86 *μ*m at the preoperative period, 279.8 ± 29.23 *μ*m at 1 month after surgery, and 274.64 ± 26.53 *μ*m at 6 months after surgery. Statistically significant differences in the average MCV and MCAT were found at 6 months after surgery (*P* = 0.008 and *P* = 0.016, resp.). In contrast, no differences were found in the average MCV and MCAT at 1 month after surgery (*P* = 0.186 and *P* = 0.318, resp.).

Preoperative and postoperative average GCIPL thickness values obtained by automated segmentation were 60.72 ± 18.20 *μ*m and 61.52 ± 17.37 *μ*m, respectively, with no statistically significant differences between the two groups (Table 2). Similarly, preoperative and postoperative average GCIPL thickness obtained by semimanual segmentation was 69.23 ± 19.50 and 65.00 ± 14.80 *μ*m, respectively, and no statistically significant differences were found either (Table 3). However, automated segmentation analysis showed statistically significant differences in the GCIPL thickness at the superior-temporal quadrant at 6 months after surgery (*P* = 0.026) (Table 4). No differences were found in the comparison of other GCIPL quadrants analyses. In contrast, when the analysis was performed by semimanual segmentation, statistically significant differences were found in the GCIPL thickness at both the superotemporal and inferotemporal quadrants at 6 months after surgery (*P* = 0.011 and *P* = 0.013, resp.) (Table 5).

Quality measurement analysis performed by automated segmentation showed that GCIPL thickness was higher in the postoperative period in around 50% of the scans (Table 6). Therefore, a significant number of segmentation errors can be expected when the automated analysis is performed in patients with macular holes. Similarly, the quality measurement analysis performed by semimanual segmentation revealed only a slight improvement in the GCIPL segmentation (Table 7).

Nowadays, ILM peeling combined with pars plana vitrectomy is considered an effective procedure for IMH surgery [1]. The removal of ILM is associated with better anatomic results and faster visual acuity recovery after surgery [1, 18, 19]. However, some adverse effects have been documented after ILM peeling, which may be associated with the use of vital dyes during surgery [20–23]. Recently, BBG-assisted ILM peeling has been reported to be safer than other dyes [24–26]. Still, a marked decrease of the average RGCC thickness at 6 months after surgery has been recently documented after ICG or BBG-assisted ILM peeling, with no differences between both dyes [17]. In contrast, Sevim and Sanisoglu showed no significant decrease of the average, superior, and inferior RGCC thickness after BBG-assisted ILM peeling [14].

The variables generated by the GCC measuring mode of the original software of the RTVue-100 SD OCT (Optovue, Fremont, CA, USA) employed by Baba et al. and Sevim et al. include the average, superior (0–180 degrees), and inferior (180–360 degrees) thickness of the RGCC, which comprises the retinal nerve fiber layer, the ganglion cell layer, and the inner plexiform layer. In contrast, the ganglion cell analysis (GCA) software of the Cirrus HD-OCT (Carl Zeiss Meditec, Dublin, CA) used in this study allows obtaining the GCIPL thickness, and therefore the retinal nerve fiber layer is not included in the measurement. Furthermore, GCA software generates specific variables for each quadrant of the macular area (superior, inferior, superonasal, inferonasal, superotemporal, and inferotemporal), as well as the average and minimum GCIPL thickness. Therefore, a more specific analysis of the inner retinal thickness area may be performed with this software. Actually, we observed no changes in the average GCIPL thickness at 1 and 6 months after surgery (Tables 3–6). However, when the analysis was performed by semimanual segmentation and individual macular quadrants, a focal GCIPL thickness decrease in the superotemporal and inferotemporal macular area was seen at 6 months after surgery (Table 5). Similarly, when the analysis was performed by automated segmentation, a focal GCIPL thickness decrease was observed in the superotemporal macular area.

Based on our results, we hypothesize that as the RNFL is thinner at the temporal area than at the nasal area [27], the ganglion cells may be more exposed to the retinal surface, and consequently to the BBG dye, which could have some toxic effects over these cells. Some in vitro studies have found a significant decrease in cell viability in both retinal pigment epithelial cells and retinal ganglion cells at exposure times to BBG as early as 3 minutes [28–30]. This reduction in cell viability has been attributed to a cytostatic effect [31]. Additionally, the ILM peeling may cause a mechanical damage over the ganglion cell layer, which is “less” protected by the RNFL at the temporal area. On the other hand, ILM was usually grasped and peeled off from the temporal quadrant, which may contribute to the mechanical damage at this area.

Several groups have reported nasal visual defects after ICG-assisted ILM peeling in IMH surgery [8–10, 32]. Some factors that may be responsible for this defect include ICG concentration, time of tissue contact, and ILM mechanical tractions [7]. However, no visual field defects have been found after BBG-assisted ILM removal [33].

The GCC color map provided by the RTVue software may allow identifying specific areas of focal ganglion cell loss. Actually, Baba et al. showed one case of a predominantly temporal GCC loss in the color-coded GCC thickness map reported [17].

On the other hand, we observed a significant decrease in the MCAT and MCV at 6 months after surgery. Some patients presented a preoperative abnormal increase of both parameters, secondary to microcystic edema usually present at the edge of the macular hole [34–36]. Additionally, MCAT and MCV results may be affected significantly by the positioning of the scanning beam in the pupil and the resultant angle of incidence on the retina [37]. In fact, this measurement error may be even more frequent when IMH is present.

**Figure 3:**
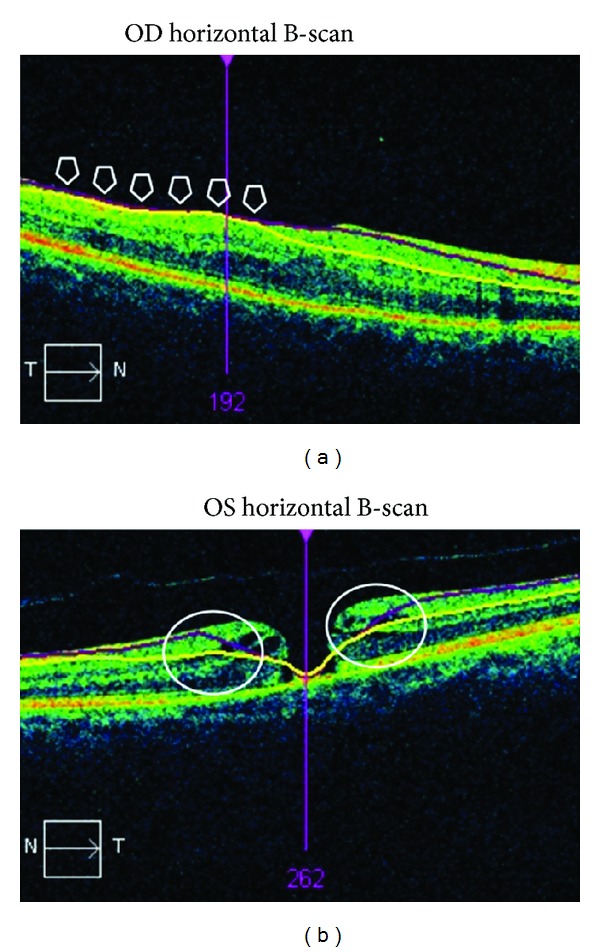
Examples of scans with incorrect ganglion cell-inner plexiform layer segmentation due to macular morphology distortion. (a) OCT ganglion cell analysis in a patient after idiopathic macular hole surgery. (b) OCT ganglion cell analysis in a patient with an idiopathic macular hole. The arrows show an area where the automated segmentation was incorrectly performed.

The study had some limitations. First, our series of patients is relatively small, in part because the GCA software of the Cirrus HD-OCT has only recently become commercially available. Therefore, future studies should be performed to validate our results. Second, longer observation periods are needed in order to evaluate the GCIPL progress over time. Third, the automatic segmentation performed by the GCA software may be altered in some patients where the macular morphology is distorted due to the IMH (Figure 3). Actually, these measurement errors may be the reason why almost no differences were found in the average GCIPL thickness in our study. In fact, we observed that GCIPL thickness analysis in some macular quadrants was higher after surgery than before surgery, even if the analysis was performed by automated or semimanual segmentation (Tables 6 and 7, resp.). Therefore, additional studies should be performed in order to determine the ability of the Cirrus HD-OCT (Carl Zeiss Meditec, Dublin, CA) to analyze the GCIPL in patients with different macular diseases. Alternatively, a real manual segmentation could be performed by custom OCT analysis software (i.e., OCTOR).

In conclusion, only focal temporal changes in the GCIPL thickness may be appreciated after vitrectomy with BBG-assisted ILM peeling for IMH. Furthermore, the results provided by Cirrus HD-OCT (Carl Zeiss Meditec, Dublin, CA) ganglion analysis software should be carefully evaluated in patients with maculopathies where there is a macular morphology distortion. However, semimanual segmentation may slightly help to improve the quality of the GCIPL analysis in these patients.

## 4. Conclusions

A significant reduction of GCIPL thickness at the temporal macular quadrants was observed by SD-OCT analysis with the new ganglion cell analysis (GCA) software of the Cirrus HD-OCT (Carl Zeiss Meditec, Dublin, CA) at 6 months after vitrectomy with BBG-assisted internal limiting membrane removal for idiopathic macular hole surgery. This reduction detection was higher when the analysis was performed by semimanual segmentation. On the other hand, an increase in the postoperative GCIPL thickness was observed in a significant number of patients, although this increase was less pronounced when the analysis was performed by semimanual segmentation. As we assume that an increase in GCIPL thickness is not expected after surgery, we hypothesize that these values may be artefacts or even a subtle inner retinal edema after ILM peeling, which could be caused by BBG dye. In this study we have evaluated the capacity of the new software of the Cirrus HD-OCT (Carl Zeiss Meditec, Dublin, CA) to analyze changes in the retinal GCIPL after internal limiting membrane peeling for idiopathic macular hole surgery, but further studies should be performed in patients with other frequent macular pathologies. To our knowledge, no study has previously validated this software in macular pathology. Glaucoma specialists must take into consideration this possible bias in GCIPL thickness analysis in patients with vitreomacular surface changes.

## Figures and Tables

**Table 1:** Demographic characteristics.

Parameter	Values	%
Sex		
Male	11	44
Female	14	56
Age∗	70.48 ± 8.66	
Eye		
Right	13	52
Left	12	48
Macular hole stage		
2	6	24
3	8	32
4	11	44
Visual acuity (log⁡MAR)		
Preoperative∗	0.70 ± 0.32	
Postoperative∗	0.34 ± 0.32	
Glaucoma		
No	25	100
Yes	0	0
Macular hole closure	25	100

*Mean ± SD.

log⁡MAR: logarithm of the minimum angle of resolution.

**Table 2:** Comparison between preoperative and postoperative (at 1 month after surgery) macular GCIPL thickness values performed by automated segmentation.

*N* = 25	Preoperative (*μ*m)	SD (*μ*m)	Postoperative (*μ*m)	SD (*μ*m)	Difference (*μ*m)	SD (*μ*m)	*P* value∗
Average GCIPL thickness	60.72	18.20	61.52	17.37	−0.8	0.83	0.865
Minimum GCIPL thickness	33.56	18.20	43.60	23.83	−10.04	−5.63	0.134
GCIPL superior	59.76	26.37	64.04	27.73	−4.28	−1.36	0.531
GCIPL inferior	53.88	23.79	59.56	18.48	−5.68	5.31	0.363
GCIPL superonasal	59.04	24.77	65.64	22.01	−6.6	2.76	0.243
GCIPL superotemporal	69.48	14.87	59.20	20.37	10.28	−5.5	0.076
GCIPL inferonasal	54.48	20.62	62.80	18.90	−8.32	1.72	0.116
GCIPL inferotemporal	67.40	20.55	59.36	22.77	8.04	−2.22	0.176

*Student's *t*-test.

GCIPL: ganglion cell-inner plexiform layer.

SD: standard deviation.

**Table 3:** Comparison between preoperative and postoperative (at 1 month after surgery) macular GCIPL thickness values performed by semimanual segmentation.

*N* = 25	Preoperative (*μ*m)	SD (*μ*m)	Postoperative (*μ*m)	SD (*μ*m)	Difference (*μ*m)	SD (*μ*m)	*P* value∗
Average GCIPL thickness	69.23	19.50	65.00	14.80	4.23	4.70	0.466
Minimum GCIPL thickness	49.69	23.29	47.85	23.78	1.85	−0.50	0.832
GCIPL superior	66.31	21.64	63.15	19.27	3.15	2.37	0.610
GCIPL inferior	63.31	22.35	63.54	16.49	−0.23	5.86	0.973
GCIPL superonasal	71.08	25.08	68.69	23.15	2.38	1.93	0.728
GCIPL superotemporal	75.23	14.21	62.08	18.27	13.15	−4.06	0.083
GCIPL inferonasal	67.15	24.13	71.69	17.07	−4.54	7.06	0.303
GCIPL inferotemporal	72.00	19.20	61.46	17.16	10.54	2.03	0.198

*Student's *t*-test.

GCIPL: ganglion cell-inner plexiform layer.

SD: standard deviation.

**Table 4:** Comparison between preoperative and postoperative (at 6 months after surgery) macular GCIPL thickness values performed by automated segmentation.

*N* = 25	Pre-operative (*μ*m)	SD (*μ*m)	Postoperative (6 m) (*μ*m)	SD (*μ*m)	Difference (*μ*m)	SD (*μ*m)	*P* value∗
Average GCIPL thickness	60.72	18.20	61.20	14.71	−0.48	3.49	0.912
Minimum GCIPL thickness	33.56	18.20	44.20	19.38	−10.64	−1.18	0.053
GCIPL superior	59.76	26.37	58.32	19.46	1.44	6.91	0.808
GCIPL inferior	53.88	23.79	60.16	15.07	−6.28	8.72	0.292
GCIPL superonasal	59.04	24.77	64.28	20.28	−5.24	4.49	0.353
GCIPL superotemporal	69.48	14.87	59.52	14.83	9.96	0.04	**0.026**
GCIPL inferonasal	54.48	20.62	64.08	15.56	−9.6	5.06	0.080
GCIPL inferotemporal	67.40	20.55	60.72	17.31	6.68	3.24	0.070

*Student's *t*-test.

GCIPL: ganglion cell-inner plexiform layer.

SD: standard deviation.

**Table 5:** Comparison between preoperative and postoperative (at 6 months after surgery) macular GCIPL thickness values performed by semimanual segmentation.

*N* = 25	Preoperative (*μ*m)	SD (*μ*m)	Postoperative (6 m) (*μ*m)	SD (*μ*m)	Difference (*μ*m)	SD (*μ*m)	*P* value∗
Average GCIPL thickness	69.23	19.50	63.77	10.14	5.46	9.36	0.241
Minimum GCIPL thickness	49.69	23.29	45.08	16.94	4.62	6.35	0.499
GCIPL superior	66.31	21.64	60.69	15.80	5.62	5.84	0.292
GCIPL inferior	63.31	22.35	62.31	14.40	1.00	7.95	0.866
GCIPL superonasal	71.08	25.08	67.85	17.23	3.23	7.85	0.590
GCIPL superotemporal	75.23	14.21	60.08	10.70	15.15	3.51	**0.011**
GCIPL inferonasal	67.15	24.13	72.54	11.13	−5.38	13.01	0.313
GCIPL inferotemporal	72.00	19.20	58.62	10.19	13.38	9.01	**0.013**

*Student's *t*-test.

GCIPL: ganglion cell-inner plexiform layer.

SD: standard deviation.

**Table 6:** Quality measurement analysis between the preoperative and postoperative macular GCIPL thickness values performed by automated segmentation.

*N* = 25	Postoperative (1 m)				Postoperative (6 m)			
	GCIPL (< or =)∗		GCIPL (>)^†^		GCIPL (< or =)∗		GCIPL (>)^†^	
Average GCIPL thickness	12	48%	13	52%	11	44%	14	56%
Minimum GCIPL thickness	9	36%	16	64%	9	36%	16	64%
GCIPL superior	11	44%	14	56%	12	48%	13	52%
GCIPL inferior	10	40%	15	60%	10	40%	15	60%
GCIPL superonasal	14	56%	11	44%	12	48%	13	52%
GCIPL superotemporal	15	60%	10	40%	18	72%	7	28%
GCIPL inferonasal	11	44%	14	56%	12	48%	13	52%
GCIPL inferotemporal	15	60%	10	40%	16	64%	9	36%
Average	**12.13**	**49%**	**12.88**	**52%**	**12.5**	**50%**	**12.5**	**50%**

*GCIPL (< or =): number of cases where the ganglion cell-inner plexiform layer thickness is lower than or equal to before surgery.

^†^GCIPL (>): number of cases where the ganglion cell-inner plexiform layer thickness is higher than before surgery.

**Table 7:** Quality measurement analysis between the preoperative and postoperative macular GCIPL thickness values performed by semimanual segmentation.

*N* = 25	Postoperative (1 m)				Postoperative (6 m)			
	GCIPL (< or =)∗		GCIPL (>)^†^		GCIPL (< or =)∗		GCIPL (>)^†^	
Average GCIPL thickness	13	52%	12	48%	14	56%	11	44%
Minimum GCIPL thickness	13	52%	12	48%	13	52%	12	48%
GCIPL superior	13	52%	12	48%	15	60%	10	40%
GCIPL inferior	14	56%	11	44%	16	64%	9	36%
GCIPL superonasal	15	60%	10	40%	14	56%	11	44%
GCIPL superotemporal	15	60%	10	40%	17	68%	8	32%
GCIPL inferonasal	12	48%	13	52%	12	48%	13	52%
GCIPL inferotemporal	13	52%	12	48%	16	64%	9	36%
Average	**13.50**	**54%**	**11.50**	**46%**	**14.63**	**59%**	**10.38**	**42%**

*GCIPL (< or =): number of cases where the ganglion cell-inner plexiform layer thickness is lower than or equal to before surgery.

^†^GCIPL (>): number of cases where the ganglion cell-inner plexiform layer thickness is higher than before surgery.
